# Relationship between vigorous physical activity and health care costs among adolescents: ABCD Growth Study

**DOI:** 10.1186/s12887-022-03201-9

**Published:** 2022-03-17

**Authors:** Wésley Torres, Lucas Gabriel de Moraes Chagas, Rômulo Araújo Fernandes, Monique Yndawe Castanho Araujo, Jacqueline Bexiga Urban, Santiago Maillane-Vanegas, Bruna Camilo Turi-Lynch, Jamile Sanches Codogno, Nana Kwame Anokye

**Affiliations:** 1 Department of Physical Education, Laboratory of InVestigation in Exercise - LIVE, São Paulo State University (UNESP), Roberto Simonsen Street, 305 Presidente Prudente, São Paulo, Brazil; 2 Department of Physical Education & Exercise Science, Lander University, Greenwood, USA; 3 Institute of Environment, Health and Societies, Brunel University, Uxbridge, UK

**Keywords:** Exercise, Pediatrics, Sedentary lifestyle, Drug costs, Health Economics

## Abstract

**Background:**

The relationship between physical activity and health care costs among adolescents is not yet clear in the literature.

**Objective:**

To analyze the relationship between physical activity and annual health care costs among adolescents.

**Methods:**

The present sample was composed of 85 adolescents of both sexes with ages ranging from 11 to 18 years (mean age 15.6 ± 2.1). Health care costs were self-reported every month for 12 months, and information on health care values was verified with local pharmacies, private health care plans, and the National Health Service. The time spent in different physical activity intensities was objectively measured by accelerometers. Confounding variables were: sex, age, somatic maturation, body fatness, blood pressure, and components of dyslipidemia and insulin resistance. Multivariate models were generated using generalized linear models with gamma distribution and a log-link function.

**Results:**

The overall annual health care cost was US$ 733.60/ R$ 2,342.38 (medication: US$ 400.46 / R$ 1,278.66; primary and secondary care: US$ 333.14 / R$ 1,063.70). The time spent in vigorous physical activity (minutes/day) was negatively related to health care costs (*r* = -0.342 [95% CI: -0.537,—0.139]; β = -0.06 cents (95% CI: -0.089, -0.031).

**Conclusion:**

Vigorous physical activity seems to be associated with lower health care costs among adolescents.

## Background

Over the last few decades, significant lifestyle changes have occurred among pediatric groups, moving toward increased time in sedentary behavior and less moderate-to-vigorous physical activity (MVPA) [1]. In terms of guidelines, the current recommendations for children and adolescents indicate that they should perform at least 60 min of MVPA daily [2]. Several health benefits are related to maintenance of adequate levels of physical activity, including improvements in cardiac and muscular outcomes [3, 4], as well as a reduced risk of obesity [5].

Although the impact of physical activity on health benefits is well known, its potential impact on economic aspects remains unclear among pediatric groups. Among adults, data from 142 countries indicate that in 2013, physical inactivity was associated with ~ US$ 54 billion of health spending worldwide [6]. Evidence also suggests that a reduction of 10% in the prevalence of physical inactivity would result in a reduction in health care expenditures of US$ 150 million per year [7].

There are plausible reasons to believe that physical activity is able to mitigate health care costs, mainly because physical activity prevents the development of many diseases and aids avoidance of early mortality in adults [8, 9]. However, among pediatric groups the issue is rarely investigated, principally because the main determinants of health care costs -chronic diseases- [10] are less common in children and adolescents. On the other hand, due to rising rates of childhood obesity and its comorbidities [11], there is a growing call for more evidence on the economic burden of these disorders among pediatric groups [12, 13], as well as for way to mitigate costs.

Although maintenance of adequate levels of physical activity among pediatric groups has proven to be effective to avoid childhood obesity and many other diseases, there is a gap in the literature regarding its impact on health care costs. Few cross-sectional studies have verified this relationship, and findings are conflicting [14, 15]. Moreover, there was sharp decline in MVPA during COVID-19 pandemic [16], reducing the amount of adolescents meeting the current physical activity recommendations [17]. Whether confirmed that higher physical activity leads to lower health care costs in adolescents, this relationship is even more concerning in a post-pandemic scenario in which a marked decline in MVPA [18, 19] prove long-lasting after schools and athletic programs resume.

Thus, the aim of the current study is to analyze the relationship between objectively measured physical activity and health care costs during one year among adolescents.

## Methods

### Sample

The cohort study entitled "Analysis of Behaviors of Children During Growth" (ABCD – Growth Study) has been ongoing in Presidente Prudente since 2017 (~ 200,000 inhabitants and human development index 0.806; western state of São Paulo, Brazil). The data came from the 2018 round of the ABCD annual survey in Brazil, which collected objective measures of physical activity, in order to identify the impact of physical activity/sports participation on different health aspects of adolescents, including health care costs.

More details about the sampling process can be found elsewhere [20, 21]. In brief, at baseline, researchers contacted adolescents in eleven school units and sports clubs spread out in the metropolitan region of the city. In the first contact with the adolescents, researchers explained all the aims and inclusion criteria of the cohort study and written consent forms were delivered to those who confirmed that they fulfilled all inclusion criteria. Inclusion criteria adopted were as follows: 1) 11–18 years old, 2) parents' consent form signed, 3) if contacted in a sports club, at least one year of training experience to characterize consistent engagement; 4) if contacted in a school unit, at least one year without regular practice of sport or exercise; 5) absence of orthopedic disease which limits physical activity.

**Fig. 1:**
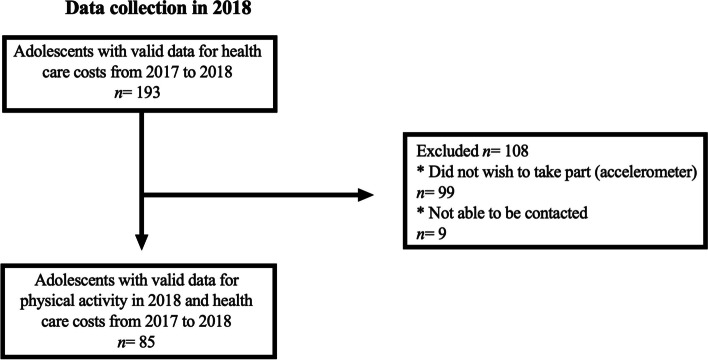
Data collection of 2018

In the second contact, researchers collected the signed written consent forms. Interviews were then scheduled by phone, to take place at the university facilities. Data collection was performed at the Laboratory of InVestigation in Exercise (LIVE) in 2017 (baseline) and 2018 (12-month follow-up). In 2018, measurements involved 193 adolescents whose health care costs were tracked from 2017 to 2018. Objective measures of physical activity were available for 85 adolescents who agreed to wear the accelerometer and had no missing data (representing 44.1% of the entire sample; ages ranging from 11 to 17 years; 58 boys and 27 girls). The flow diagram of the study is presented in Fig. 1. Adolescents who wore the accelerometer (*n* = 85) and adolescents who did not (*n* = 108) were similar in terms chronological age (*p*-value = 0.87), somatic maturation (*p*-value = 0.62), birth weight (*p*-value = 0.52), LDL-C (*p*-value = 0.26), HDL-C (*p*-value = 0.69), HOMA-IR (*p*-value = 0.06), SBP (*p*-value = 0.08), DBP (*p*-value = 0.32), and RHR (*p*-value = 0.89). Moreover, sex distribution was similar in both groups (*p*-value = 1.00).

The authors defined the relationship between physical activity and health care costs as the main analysis of the current work, and, therefore, the sample size calculation was based on measures of relationship, in this case correlation (*r*), mainly because correlations are easy to interpret measures of effect-size [22]. The statistical equation adopted to estimate the sample size considered a statistical power of 80% and a significance of 0.05 (Z = 1.96) [23]. Thus, our sample size of 85 adolescents was able to detect coefficients of correlation ≥ 0.30, being less effective to detect significant relationships of small magnitude.

## Ethical approval

All methods were carried out in accordance with relevant guidelines and regulations along with an ethical approval statement and informed consent to participation according to the Declaration of Helsinki. Ethical approval was obtained from the Ethics Research Committee of the Sao Paulo State University (process number: 1.677.938/2016), campus of Presidente Prudente, State of Sao Paulo, Brazil. Adolescents whose parents / legal guardians provided signed informed consent were eligible for participation. A letter with information about the study was provided for participants and was explained to eligible participants by trained researchers. Adolescents could at any time quit the study without negative consequences. There were no incentives for participation.

## Dependent variable: health care costs

As there are no previously validated methods to assess health care costs among children and adolescents, our study adopted an approach previously established to assess this outcome in adults [24–26]. Participants received a log to register all health care events (e.g. medical consultations, hospitalizations, laboratory tests, buying medicines), after receiving appropriate training on how to complete the logs from the researchers. Researchers contacted the participants (adolescents and their parents/legal guardians) monthly by phone to confirm the data registered in the logs. The logs contained data about the use of medication (name and dosage) and primary and secondary care (medical specialties [e.g. pediatrician, general practitioner, ophthalmologist, orthopedist, ear, nose and throat specialist, dermatologist, pulmonologist, emergency doctor, physical therapist, speech therapist, psychotherapist, occupational therapist, homeopath, and others], occurrence of hospitalizations, laboratory tests, and other occurrences [e.g. car accidents]). Moreover, the log contained fields for data about the way the medical service had been paid (private health care plans, National Health Service, and out-of-pocket costs). After collecting the data registered in the logs, the researchers contacted pharmacies (when bought by the participant), private health care plans (when paid by the participant), and the National Health Service (when provided by the government) for 12 months to check the price of all health care services. The prices paid by the National Health Service were provided by the local authorities (Department of Health), while the prices paid by private health care plans were established through direct consultation with the plans. Regarding medicines bought by the participants in pharmacies, we generated a weighted average unit price considering three different pharmacies (price collected by three independent researchers). There were no occurrences of health care costs attributed to hospitalizations and surgeries in the current study (only primary care services were reported by the participants). Moreover, the health care costs of all 85 adolescents were assessed monthly with no missing data.

The prices were estimated in the Brazilian currency (Real [R$]), and then converted into American dollars (US$). The exchange rate used was the average quotation (R$ versus US$) of the 12 months of follow-up (from 2017 to 2018 [US$ 1.00 is equal to R$ 3.193]), which was provided by the Brazilian Federal Reserve [27]. In addition, the health care costs were updated by the inflation observed in 2019 and 2020 (accumulated until September) [27].

## Independent variable: objectively measured physical activity

Physical activity level and sleep were collected using an accelerometer (Polar V800; Polar, Kempele, Finland). The Polar V800 provides accurate data about physical activity when compared to other accelerometers [28]. However, data on epoch length, definition of non-wearing time, and cut-off points for counts are pre-fixed by the manufacturer and not available in the manual (and were not released even after requested by the authors) [29]. The Polar V800 is a sport watch with an internal tri-axial accelerometer that records wrist movements (dimensions of 37 mm × 56 mm × 12.7 mm; weight of 79 g) [30].

The participant wore the device for five consecutive weekdays. The first and last days were excluded and thus three valid weekdays were counted (at least one weekend day) [31, 32]. The device is waterproof and provides data about sleeping time. Adolescents wore the accelerometer for the entire day, except during sports participation due to the risk of injuries (as requested by the coaches). For each day analyzed, the device discriminated: I) step count, II) sleep time, III) rest time (sleep and rest, lying down), IV) sitting time (sitting and other passive behaviors), V) light (standing activities, light intensity tasks), VI) moderate (walking and other moderate activities), and VII) vigorous intensity activities (jogging, running, and other intense activities) [29]. The average time of the three valid weekdays was counted and adolescents were classified as sufficiently (≥ 60 min/day of moderate-to-vigorous [VI + VII] physical activity [MVPA]) or insufficiently active (< 60 min/day of MVPA) [33].

## Confounders

It was assumed that obesity, sex, age, birth weight, biological maturation, and cardiovascular and metabolic parameters could be potential correlates among adolescents. The majority of these variables are correlates of health care costs in adults [24–26], except biological maturation and birth weight. In a face-to-face interview, the adolescents reported sex and birth date (chronological age), and parents/legal guardians provided data about birth weight. Somatic maturation (age at peak of height velocity [PHV]) was estimated using anthropometric measures [34], while whole body fatness (in percentage [BF%]) was assessed using a dual-energy x-ray absorptiometry (DXA) scanner (Lunar DPX-NT; General Electric Healthcare, Little Chalfont, Buckinghamshire, UK) with GE Medical System Lunar software (version 4.7).

The self-report of musculoskeletal symptoms in any of nine body segments (neck, shoulder, upper back, low back, elbows, wrists/hands, hips/thighs, knees, and ankles/feet) in the previous week (before the face-to-face interview) was assessed using the Nordic Musculoskeletal Questionnaire [35]. A continuous variable considering the number of positive answers was created (score ranging from 0 [no symptoms reported] to 9 [symptoms reported in all body segments]).

Cardiovascular measures were taken three times after 10 min resting. Resting heart rate (RHR), and systolic (SBP) and diastolic blood pressure (DBP) were measured using electronic devices (Omron Healthcare, Inc., Intellisense, model HEM 742 INT, Bannockburn, Illinois, USA), validated for the pediatric population [36]. The blood samples were collected with the patient sitting comfortably in their own chair, with arm supported and elbow straight, 10 mL of fasting venous blood were collected from the elbow crease in the morning by a trained nurse. High-density lipoprotein [HDL-C], low-density lipoprotein [LDL-C], glucose, and insulin were measured in order to calculate the homeostatic model assessment [HOMA-IR]), and a private laboratory was responsible for all laboratory procedures (this laboratory meets all quality standards established by the Brazilian Ministry of Health).

## Statistical analysis

Continuous data are expressed as mean and 95% confidence interval (95%CI). Median, 25^th^ percentile (P25^th^) and 75^th^ percentile (P75^th^) were used because health care costs and physical activity showed non-normal distribution. Bivariate analyses used the Mann–Whitney test to compare health care costs according to physical activity level (sufficiently active versus insufficiently active). The Pearson correlation and its 95%CI were used to assess the crude relationship between health care costs and physical activity. Health care costs were converted into log-10 due to non-parametric distribution. Generalized linear models using gamma distribution with a log-link function ([GLM-Gamma], expressed as β and its 95%CI) were employed to examine the relationship between physical activity and healthcare costs, controlling for covariates (sex, age, somatic maturation, birth weight, body fatness, resting heart rate, low and high density lipoprotein, musculoskeletal symptoms, and systolic and diastolic blood pressure). Due to the non-parametric distribution of the health care costs, a general linear model conserving a Gamma error distribution with a log link (GLM-Gamma) was adopted as the multivariate model, as it fits well for positive-only data with positively-skewed errors [37]. The GLM-Gamma model was run considering a hierarchical approach to insert covariates, as follows: Model-1 (crude); Model-2 (adjusted by general information [sex, age, biological maturation, birth weight, and adiposity]); Model-3 (Model-2 plus metabolic variables [LDL-C, HDL-C, and HOMA-IR]); Model-4 (Model-3 plus cardiovascular variables [SBP, DBP, and RHR] and musculoskeletal symptoms). Statistical significance was set at p < 0.05 and all analyses were performed using BioEstat software (version 5.2 [BioEstat, Teffe, Brazil]).

## Results

The sample was composed of 85 adolescents with ages ranging from 11 to 17 years (mean age of 15.6 ± 2.1) and predominately males (68.2% [*n* = 58] versus 31.8% [*n* = 27], respectively; *p*-value = 0.01). The proportion of adolescents meeting the physical activity guidelines was similar between boys and girls (40.9% [*n* = 49] versus 34.6% [*n* = 36], respectively; *p*-value = 0.78).

Considering the entire sample, overall annual health care costs were US$ 733.60/ R$ 2,342.38 (medication: US$ 400.46 / R$ 1,278.66; primary and secondary care: US$ 333.14 / R$ 1,063.70). For medication, 27 adolescents reported the use of 50 drugs (18 paid for by the National Health Service, 31 out-of-pocket, and 01 by a private health care plan). For primary and secondary care, 17 adolescents reported 19 medical consultations (10 paid for by the National Health Service, 02 out-of-pocket, and 07 by private health care plans), being *n* = 07 general practitioner, *n* = 05 orthopedist, *n* = 02 pediatrician, *n* = 02 dermatologist, *n* = 01 pulmonologist, *n* = 01 neurologist, and *n* = 01 cardiologist. Moreover, nine adolescents reported 10 tests (4 paid for by the National Health Service and 6 by private health care plans), being *n* = 05 X-ray (chest, foot, and hand), *n* = 03 ultrasound (hand, knee, and abdomen), *n* = 01 blood test, and *n* = 01 electrocardiogram.

Adolescents meeting the physical activity guidelines presented similar values of somatic maturation, body composition, blood pressure, and birth weight to those adolescents who did not meet the guidelines. On the other hand, the time in a sitting position was 14% lower among adolescents meeting the physical activity guidelines compared to those who did not (496.7 min/day [P25^th^-P75^th^: 426.1 to 531.1] versus 578.0 min/day [P25^th^-P75^th^: 494.1 to 637.8], respectively) (Table 1).

**Table 1:** General characteristics of the adolescents according to the physical activity recommendations (*n* = 85)

	MVPA (< 60 min/day) (*n* = 49)	MVPA (≥ 60 min/day) (*n* = 36)
Variables	Mean (95%CI)	Mean (95%CI)
Boys / Girls	33 / 16	25 / 11
Age (years)	15.7 (15.1 to 16.2)	15.6 (14.7 to 16.5)
Body weight (kg)	62.5 (58.1 to 66.9)	66.4 (60.6 to 72.1)
Height (cm)	169.2 (166.5 to 171.1)	170.9 (167.2 to 174.6)
PHV (years)	1.9 (1.5 to 2.3)	2.1 (1.4 to 2.7)
Birthweight (g)	3234.5 (3084.9 to 3247.8)	3227.8 (2978.4 to 3477.3)
BF (%)	21.6 (18.4 to 24.8)	22.5 (18.7 to 26.3)
LDL-C (mg/dL)	74.6 (68.2 to 81.1)	67.2 (61.7 to 72.6)
HDL-C (mg/dL)	53.2 (49.7 to 56.7)	50.8 (47.2 to 54.5)
HOMA-IR	2.1 (1.5 to 2.5)	1.9 (1.5 to 2.2)
SBP (mmHg)	115.9 (112.7 to 119.2)	119.2 (114.2 to 124.2)
DBP (mmHg)	64.5 (62.5 to 66.5)	66.1 (63.2 to 68.8)
RHR (bpm)	75.2 (72.3 to 78.1)	73.6 (70.1 to 77.2)
	Median (P25^th^ – P75^th^)^a^	Median (P25^th^ – P75^th^)^a^
Not wearing (min/day)	17.3 (0.0 to 57.3)	7.1 (0.0 to 37.5)
Sitting position (min/day)	578.0 (494.1 to 637.8)	496.7 (426.1 to 531.1)
Light PA (min/day)	261.1 (226.3 to 303.5)	319.7 (269.1 to 379.1)
Moderate PA (min/day)	33.7 (23.5 to 41.1)	69.8 (57.9 to 81.2)
Vigorous PA (min/day)	4.1 (1.3 to 7.1)	15.1 (6.7 to 23.3)
Sleeping time (min/day)	401.7 (367 to 484.8)	461.3 (413.1 to 484.3)
MKS (score)	1.0 (0.0 to 2.0)	1.0 (0.0 to 3.0)

*MVPA* moderate-to-vigorous physical activity, *PA* physical activity, *SD* standard-deviation, *PHV* peak of height velocity (somatic maturation), *BF* body fatness by DXA, *SBP* systolic blood pressure, *DBP* diastolic blood pressure, *RHR* resting heart rate, *LDL-C* low-density lipoprotein, *HDL-C* high-density lipoprotein, *HOMA-IR* Homeostatic model assessment, *MKS* musculoskeletal symptoms

^a^variable expressed as median, percentile 25^th^ and percentile 75^th^ due to non-normal distribution (comparisons performed using Mann–Whitney’s test),

**Fig. 2:**
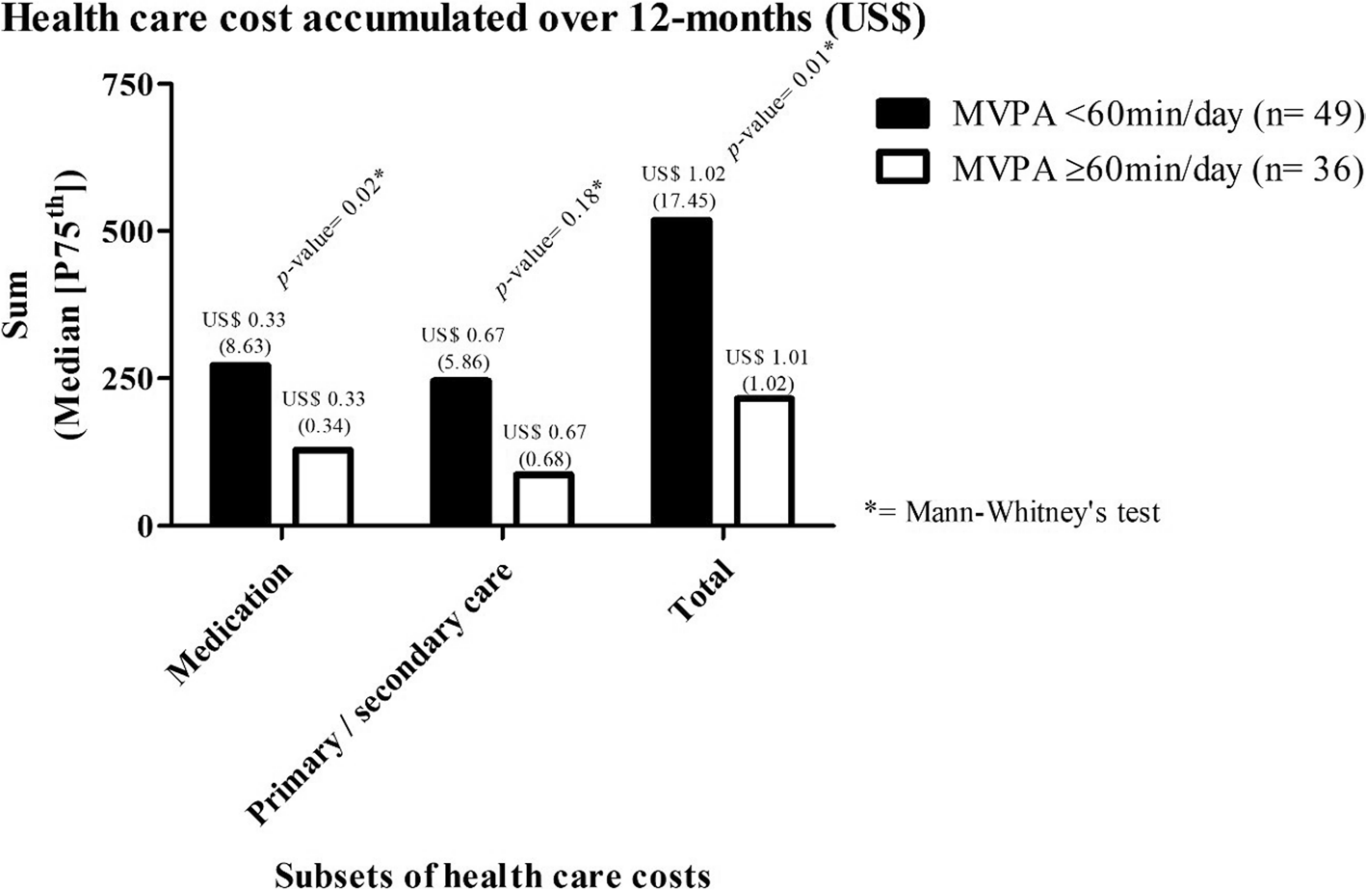
Health care cost accumulated over 12 months (US$)

Adolescents who reached the physical activity recommendations, when compared to those adolescents who did not, accumulated less health care costs for medication (Median = US$ 0.33 [P75^th^: 0.34] versus Median = US$ 0.33 [P75^th^: 8.63], respectively) and total health care costs (Median = US$ 1.01 [P75^th^: 1.02] versus Median = US$ 1.02 [P75^th^: 17.45], respectively) (Fig. 2).

Considering all possible intensities of physical activity and their relationship with the health care costs over the previous year (Table 2), the time spent in vigorous physical activity was negatively related to health care costs with a small magnitude (*r* = -0.342; 95%CI: -0.537, -0.139]).

**Table 2:** Relationship between health care costs, covariates, and physical activity among adolescents (*n* = 85)

	Dependent variable: Overall health care costs (US$)
	Pearson correlation
Independent variables	*r*_crude_ (95%CI)
General	
Sex (girl = 0 / boy = 1)	-0.017 (-0.229 to 0.197)
Age (years)	0.092 (-0.124 to 0.299)
PHV (years)	0.202 (-0.012 to 0.398)
Birthweight (g)	0.026 (-0.188 to 0.238)
BF (%)	0.028 (-0.186 to 0.240)
LDL-C (mg/dL)	0.290 (0.082 to 0.474)*
HDL-C (mg/dL)	0.052 (-0.163 to 0.262)
HOMA-IR	-0.105 (-0.311 to 0.111)
SBP (mmHg)	-0.206 (-0.402 to 0.007)
DBP (mmHg)	-0.246 (-0.436 to -0.035)*
RHR (bpm)	0.081 (-0.134 to 0.289)
MKS (score)	-0.065 (-0.274 to 0.150)
Accelerometer	
Seating position (min/day)	0.132 (-0.083 to 0.336)
Light PA (min/day)	0.146 (-0.069 to 0.348)
Moderate PA (min/day)	-0.200 (-0.396 to 0.014)
Vigorous PA (min/day)	-0.342 (-0.517 to -0.139)*
Sleeping time (min/day)	-0.130 (-0.334 to 0.085)

*PA* physical activity, *95%CI* 95% confidence interval, *PHV* peak of height velocity (somatic maturation), *BF* body fatness by DXA, *SBP* systolic blood pressure; *DBP* diastolic blood pressure, *RHR* resting heart rate, *LDL-C* low-density lipoprotein, *HDL-C* high-density lipoprotein, *HOMA-IR* Homeostatic model assessment, *MKS* musculoskeletal symptoms

* = p-value < 0.05

In the GLM-Gamma (Table 3), vigorous physical activity was negatively related to total health care costs (US$ -0.06 [95%CI: -0.089 to -0.031] / R$ -0.19 [95%CI: -0.28 to -0.10]), independently of confounders. Among these adolescents, the LDL-C covariate was positively related to total health care costs (β = 0.015 [95%CI: 0.001 to 0.029]).

**Table 3:** Multivariate relationship between health care costs and physical activity among adolescents (*n* = 85)

	GLM—Gamma (dependent variable: overall health care costs [US$])			
	Model—1	Model—2	Model—3	Model—4
Independent variable	(β _95%CI_)	(β _95%CI_)	(β _95%CI_)	(β _95%CI_)
Vigorous PA (min/day)	-0.073(-0.097; -0.048)*	-0.063(-0.089; -0.036)*	-0.059(-0.087; -0.031)*	-0.060(-0.089; -0.031)*
Cofounders				
Sex (girl = 0 / boy = 1)		0.001 (-0.773; 0.774)	-0.393 (-1.322; 0.536)	-0.492 (-1.667; 0.683)
Age (years)		-0.492(-0.907; -0.077)*	-0.296 (-0.752; 0.160)	-0.318 (-0.774; 0.139)
PHV (years)		0.695 (0.175; 1.214)*	0.400 (-0.185; 0.984)	0.372 (-0.273; 1.016)
Birthweight (kg)		-0.159 (-0.547; 0.229)	-0.012 (-0.479; 0.455)	-0.079 (-0.562; 0.405)
BF (%)		-0.015 (-0.044; 0.015)	-0.024 (-0.061; 0.13)	-0.029 (-0.072; 0.015)
LDL-C (mg/dL)			0.014 (0.001; 0.028)*	0.015 (0.001; 0.029)*
HDL-C (mg/dL)			-0.012 (-0.039; 0.014)	-0.015 (-0.044; 0.014)
HOMA-IR			0.030 (-0.209; 0.270)	0.053 (-0.229; 0.335)
SBP (mmHg)				0.010 (-0.018; 0.037)
DBP (mmHg)				0.305 (-0.692; 1.305)
RHR (bpm)				-1.369 (-3.825; 1.101)
MKS (score)				0.004 (-0.169; 0.177)

*GLM* generalized linear model, *PA* physical activity, *95%CI* 95% confidence interval, *PHV* peak of height velocity (somatic maturation), *BF* body fatness by DXA, *SBP* systolic blood pressure, *DBP* diastolic blood pressure, *RHR* resting heart rate, *LDL-C* low-density lipoprotein, *HDL-C* high-density lipoprotein, *HOMA-IR* Homeostatic model assessment, *MKS* Musculoskeletal symptoms

* = p-value < 0.05

## Discussion

In the current study, adolescents who met the general recommendations for physical activity accumulated less health care costs during 12 months of follow-up compared to those who did not, mainly due to the time spent in vigorous activities.

The results showed that physical activity and health care costs were inversely related. This theme is surrounded by uncertainty in the pediatric literature (e.g. the existence of the relationship and even its direction), mainly because although this research topic has received growing attention recently, the focus has been on adults [6]. A previous German survey evaluated more than 3,000 children aged 9 to 12 years and failed to find a significant relationship between physical activity and health care costs [38], concluding that the moment of childhood is an early stage to determine significant effects of physical activity on health expenditures.

On the other hand, differences in methodology might justify these divergences from the literature. First, in our study, health care costs attributed to medication were more prone to be inversely related to physical activity. Medication accounted for 54.5% of all health care costs in our sample, while the study by Idler et al. [15] counted health care costs for primary and secondary care but not medication. Similarly, the inverse relationship between physical activity (number of steps) and costs attributed to medicine has also been identified in 40–65 year-old adults [39].

Second, the way in which physical activity was assessed in both studies deserves to be highlighted. In the German study, physical activity was self-reported using a questionnaire [15, 38], while in our study physical activity was assessed using an objective measurement method (accelerometers). This technical issue seems more relevant to support the differences between both studies because only vigorous physical activity was inversely related to economic variables. In fact, although questionnaires are useful in large surveys, they commonly fail to precisely record the time spent in different physical activity intensities [40]. Therefore, if the main pathway linking physical activity and health care costs in adolescents is the intensity, the limitation of questionnaires becomes critically relevant.

Initially, it was supposed that obesity would be the main factor supporting an inverse relationship between physical activity and health care costs among these adolescents [12, 13]. However, our findings refuted that hypothesis, in which higher values of LDL-C assumed a relevant role as a determinant of health care costs, independently of physical activity. First, the independence between both is justified because, as mentioned above, physical activity seems to be particularly related to health care costs attributed to medication, while LDL-C values were related to health care costs attributed to primary and secondary care (*r* = 0.291; *p*-value = 0.009). In fact, dyslipidemia is a health outcome with growing prevalence among adolescents (reaching 61% depending on the cut point adopted) [41] and, naturally, leading to a higher number of medical consultations in order to treat it. Moreover, even assuming that alterations in LDL-C concentration are strongly affected by obesity, this is not mandatory, because dyslipidemia also occurs in children and adolescents with normal weight but with poor diet habits [42].

In terms of limitations, it is necessary to recognize some in our study. First, although all health care costs were counted over a 12-month period, physical activity was measured from a cross-sectional perspective (only in the follow-up moment). Therefore, it is desirable that future studies try to identify the impact of changes in physical activity on health care costs, mainly because our measure of physical activity does not represent the whole period of follow-up. Moreover, sports participation is the main manifestation of physical exercise in adolescence (relevant source of vigorous physical activity) and its absence from the step counts is worth mentioning. Second, even considering that health care costs were self-reported every single month, it is necessary to consider the presence of recall bias in the information reported by parents and adolescents, leading to possible underestimation of the real monetary values. Third, the small sample size is a limitation in this study due to obvious issues related to the generalization of the findings. In adults, physical activity intensity and health care costs from primary care are significantly related to each other [43], but in a smaller magnitude (*r* = -0.22) than our sample size was able to detect (*r* = -0.30). The fact our sample size was small might have affected the ability to detect the relationship between health care costs and other domains of physical activity intensity (e.g. light and moderate). Therefore, while vigorous physical activity should be recommended for pediatric groups in order to promote health and mitigation of health care costs [44], other activities requiring less energy expenditure should also be recommended. Finally, data on epoch length, definition of non-wearing time, and cut-off points for counts were pre-fixed by the manufacturer and were not available to the research team. The absence of these data is relevant because it limits comparisons with previous studies using accelerometers (due to the number of cut-points adopted in the literature).

The period of time assessed in our study does not cover the COVID-19 pandemic, but our findings highlight a concerning post-pandemic scenario. Studies around world, indicate that the amount of time dedicated to MVPA dropped around 30% in adolescents due to social distancing measures [16], while the percentage of engagement in physical activity ≥ 5 days/week decreased 22% during COVID-19 pandemic (from 50 to 39%) [17]. Whether pandemic related declines in MVPA prove long-lasting may lead to long-term health implications with substantial economic impact. Therefore, in the post-pandemic world where economies were severely affected by social distancing measures, governmental actions targeting the promotion of MVPA in children and adolescents could be a powerful tool for both promoting health and for minimizing health care spending.

## Conclusion

Our findings suggest that only vigorous physical activity seems to be associated with lower health care costs among adolescents, denoting the relevance of the regular engagement of adolescents in activities of higher physical demand.

## Data Availability

The data collected and analyzed during this study are stored by the authors upon authorization by the leader of the Laboratory of InVestigation in Exercise (LIVE) which involves the ABCD Growth Study.

## Abbreviations

MVPA: Moderate-to-vigorous physical activity
ABCD Growth Study: Analysis of Behaviors of Children During Growth
UNESP: São Paulo State University
LIVE: Laboratory of InVestigation in Exercise
LDL-C: Low-density lipoprotein
HDL-C: High-density lipoprotein
HOMA-IR: Homeostatic model assessment of insulin resistance
SBP: Systolic blood pressure
DBP: Diastolic blood pressure
RHR: Resting heart rate
PHV: Peak height velocity
BF: Body fatness
DXA: Dual-energy x-ray absorptiometry
